# New insights in the management of Hepatocellular Adenoma

**DOI:** 10.1111/liv.14547

**Published:** 2020-06-11

**Authors:** Anne J. Klompenhouwer, Robert A. de Man, Marco Dioguardi Burgio, Valerie Vilgrain, Jessica Zucman‐Rossi, Jan N. M. Ijzermans

**Affiliations:** ^1^ Department of Surgery Erasmus MC University Medical Center Rotterdam the Netherlands; ^2^ Department of Gastroenterology and Hepatology Erasmus MC University Medical Center Rotterdam the Netherlands; ^3^ Department of Radiology Hauts‐de‐Seine University Hospitals Paris Nord Val de Seine Beaujon, APHP Clichy France; ^4^ Centre de Recherche sur l'inflammation (CRI) INSERM U1149 et Université de Paris Paris France; ^5^ Centre de Recherche des Cordeliers Sorbonne Université, INSERM Université de Paris Paris France; ^6^ Oncology Department APHP Hôpital européen Georges Pompidou Paris France

**Keywords:** Hepatocellular Adenoma, management, review

## Abstract

Hepatocellular adenoma (HCA) are benign liver tumours that may be complicated by haemorrhage or malignant transformation to hepatocellular carcinoma. Epidemiological data are fairly outdated, but it is likely to assume that the incidence has increased over the past decades as HCA are more often incidentally found due to the more widespread use of imaging techniques and the increased incidence of obesity. Various molecular subgroups have been described. Each of these molecular subgroups are defined by specific gene mutations and pathway activations. Additionally, they are all related to specific risk factors and show a various biological behaviour. These molecular subgroups may be identified using immunohistochemistry and molecular characterization. Contrast‐enhanced MRI is the recommended imaging modality to analyse patients with suspected hepatocellular adenoma allowing to determine the subtype in up to 80%. Surgical resection remains to be the golden standard in treating HCA, although resection is deemed unnecessary in a large number of cases, as studies have shown that the majority of HCA will regress over time without complications such as haemorrhage or malignant transformation occurring. It is preferable to treat patients with suspected HCA in high volume centres with combined expertise of liver surgeons, hepatologists, radiologists and (molecular) pathologists.

**Key points**

- Epidemiological data on HCA are fairly outdated but it is likely to assume that the incidence has increased over the past decades as HCA are more often incidentally found due to the more widespread use of imaging techniques and the increased incidence of obesity.
- Various molecular subgroups of HCA have been described with varying biological behaviour.
- These molecular subgroups may be identified using contrast‐enhanced MRI, immunohistochemistry and molecular characterization.
- Surgical resection remains to be the golden standard in treating HCA, although resection is deemed unnecessary in a large number of cases.
- As HCA may be considered a rare disease, it is preferable to treat patients with suspected HCA in high volume centres with combined expertise of liver surgeons, hepatologists, radiologists and (molecular) pathologists.

## 1 INTRODUCTION

Hepatocellular adenoma (HCA) is a benign liver tumour that may be complicated by haemorrhage or malignant transformation to hepatocellular carcinoma (HCC). Risk factors for HCA include long‐term use of the oral contraceptive pill (OC),^1^, ^2^ obesity and the metabolic syndrome,^3^, ^4^, ^5^ androgen consumption ^6^ and genetic disorders such as MODY‐3 and glycogen storage disease.^7^, ^8^, ^9^ Two clinical practice guidelines have been issued on the management of benign liver tumours: the first from the American College of Gastroenterology (ACG, dating from 2014) and the second from the European Association for the Study of the Liver (EASL, dating from 2016).^10^, ^11^ Since the publication of these guidelines, much progress has been made in the field of hepatocellular adenoma. In this review, we describe the major recent advances in this field, including epidemiology, diagnosis (imaging and pathology), prognosis and treatment and discuss the implications in clinical practice.

## 2 EPIDEMIOLOGY

For a long time, epidemiological data on HCA were severely outdated. In 1979, the annual incidence rate was estimated at 3‐4 per 100.000 women per year for long‐term OC users, as compared to 0.1 per 100.000 women per year for non‐long‐term users.^12^ The next study concerning the epidemiology of HCA was not published until 2017. This was a nationwide registry‐based cohort study from Denmark.^13^ The authors investigated the incidence of hepatocellular adenoma and found a standardized incidence rate of biopsy‐confirmed adenomas of 0.07 per 100.000 population per year (0.02 per 100.000 for men and 0.13 for women). The true incidence rate, however, will probably be higher as only patients with biopsy‐confirmed HCA were included in this study. It is likely to assume that the incidence has increased since 1979 as HCA are more often incidentally found due to the more widespread use of imaging techniques. Additionally, the obesity epidemic may have led to an increase in the incidence of HCA.^3^, ^4^, ^14^ Epidemiological data on HCA from Europe and the United States have seldom been compared to data from continents where both the use of oral contraceptives and the incidence of obesity is lower. A recent single‐centre study from Taiwan showed that the local incidence of HCA increased over the last decade and that the clinical features differ from those reported in Europe and the United States.^15^ For instance, they found a male predominance in their cohort. It would be very interesting to further explore these differences in epidemiological data.

## 3 PATHOLOGY

HCA results from a monoclonal benign proliferation of hepatocytes. Usually, tumour hepatocytes in HCA look similar to normal hepatocytes. During the last 20 years, genomics analyses of large series of hepatocellular adenomas enabled to progressively identify six major molecular subgroups and additional mixed subtypes of HCA. Each of these molecular subgroups are defined by specific gene mutations and pathway activations. They are all related to specific risk factors and show a various biological behaviour.

### 3.1 HNF1A inactivated HCA (H‐HCA)

The first molecular subgroup accounts for 30% to 40% of adenomas and is defined by a mutation inactivating HNF1A, a gene coding for Hepatocyte Nuclear Factor 1 Alpha. This is a transcription factor essential for the differentiation of hepatocytes.^16^ HNF1A inactivation in hepatocytes leads to several metabolic alterations with an activation of lipogenesis.^17^, ^18^ As a result, HNF1A inactivated adenomas accumulate lipids in tumour hepatocytes leading to a characteristic homogeneous steatotic phenotype at histology, without inflammatory infiltrates. Multiple H‐HCA is frequent in patients, and it is often referred to liver adenomatosis when more than 10 H‐HCA are identified in the liver. In rare cases, familial liver adenomatosis has been described related to a Maturity Onset Diabetes type 3 (MODY3) with a transmitted HNF1A germline mutation.^7^, ^19^

### 3.2 Inflammatory HCA (I‐HCA)

I‐HCA is the most frequent subtype (40%‐50% of the cases), defined by the activation of STAT3, a major transcription factor of inflammation. In I‐HCA, an activating mutation of one the factors of the IL6/STAT3 signalling pathway is identified targeting either IL6ST, FRK, STAT3, JAK1, GNAS1 or ROS1.^20^, ^21^, ^22^ Each of these gene activations leads to the overexpression of the proteins of the acute inflammatory phase including SAA (serum amyloid protein) and CRP (C‐reactive protein), by tumour hepatocytes. At histology, I‐HCAs show marked inflammatory infiltrates together with a high vascularization combining small arteries and telangiectasia. Immunohistochemistry shows a typical staining of tumour hepatocytes using antibodies against SAA or CRP. I‐HCA is frequently identified in obese patients and can be associated with alcohol intake. They are also frequent in patients with vascular liver diseases and finally multiple I‐HCA and liver I‐HCA adenomatosis are described.^19^, ^21^

### 3.3 Beta‐catenin activated HCA (b‐HCA)

ß‐catenin is an important oncogene in the liver. Two types of mutations in the CTNNB1 gene have been identified in HCA, leading to an activation of ß‐catenin.

Mutations or deletions at exon 3 of CTNNB1 are identified in 10 to 15% of HCA (b^ex3^HCA). These alterations are well‐known oncogenic mutations, that lead, in the vast majority of the cases, to a high activation of the WNT/ß‐catenin pathway.^23^, ^24^ The histological phenotype of the tumour usually combines tumour cholestasis, cytological atypia and tumour dysplasia. Using immunohistochemistry, a homogeneous overexpression of GS (glutamine synthetase) is detected with a nuclear accumulation of ß‐catenin in some cases. This subtype is frequently associated with a malignant transformation to hepatocellular carcinoma, particularly in males.^21^, ^23^

Around 7 to 10% of HCA show atypical hotspot mutation at exon 7 or 8 of CTNNB1 (b^ex7,8^HCA).^25^ These mutations lead to a faint ß‐catenin activation, and immunohistochemistry show only a heterogeneous and weak expression of glutamine synthetase without nuclear ß‐catenin. Interestingly, malignant transformation to hepatocellular carcinoma has been described far less in CTNNB1 mutations in exon 7 or 8 as compared to exon 3.^26^ Overall, b‐HCA is not steatotic and do not show inflammatory infiltrates.^21^, ^25^

### 3.4 Sonic hedgehog activated HCA (sh‐HCA)

In 5% of all hepatocellular adenomas, an activation of GLI1, a major transcription factor of the sonic hedgehog pathway, is observed. As a consequence, sh‐HCA shows an overexpression of specific genes such as PTGDS (Prostaglandin D2 Synthase) that can be demonstrated by immunohistochemistry.^21^ Sh‐HCA is frequently identified in obese patients and they are associated with a higher risk of bleeding. However, currently no other specific histological features are associated with this subtype.

### 3.5 Mixed beta‐catenin‐inflammatory adenoma (b‐IHCA)

Mixed molecular subclasses showing both inflammatory and ß‐catenin activation and resulting in b^ex3^IHCA or b‐^ex7,8^IHCA are observed in around 10% of the cases. Their histological pattern results from the combination of each subtype. In contrast, H‐HCA or sh‐HCA is almost never mixed with another molecular subtype.^21^

### 3.6 Unclassified HCA (U‐HCA)

**FIGURE 1:**
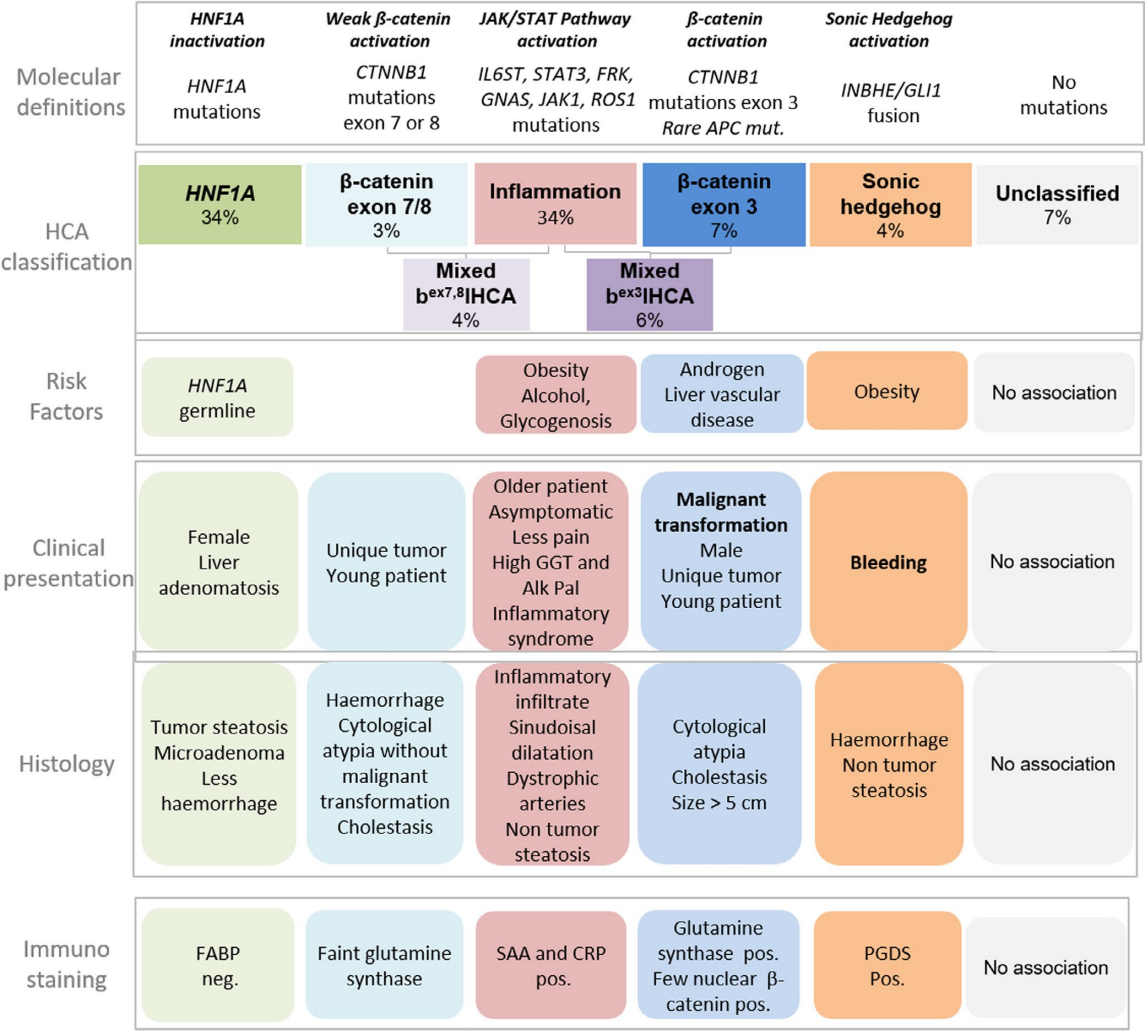
Overview of HCA subtypes

Overall, in less than 7%, HCA remain unclassified. They do not show specific histological features. An overview of all molecular subtypes, risk factors and biological behaviour is given in Figure 1.

## 4 IMAGING

As suggested by the 2016 EASL guidelines on the management of benign liver tumours, contrast enhanced MRI is the recommended imaging modality to analyse patients with suspected HCA allowing to determine the subtype in up to 80%.^11^ Additionally, MRI is useful to differentiate HCA from focal nodular hyperplasia (FNH) using the combination of classical diagnostic criteria for FNH and lesion behaviour on hepatobiliary phase MRI using liver‐specific contrast agents.^27^ Contrast‐enhanced CT also allows the visualization of lesion enhancement patterns and dilatation of intratumoral sinusoids, without any additional information over MRI. Contrast‐enhanced ultrasound can be performed in addition to MRI to differentiate small (<3 cm) HCA from FNH in doubtful cases, although it has a limited value in differentiating among different HCA subtypes.^11^, ^28^, ^29^

### 4.1 HNF1A inactivated HCA

**FIGURE 2:**
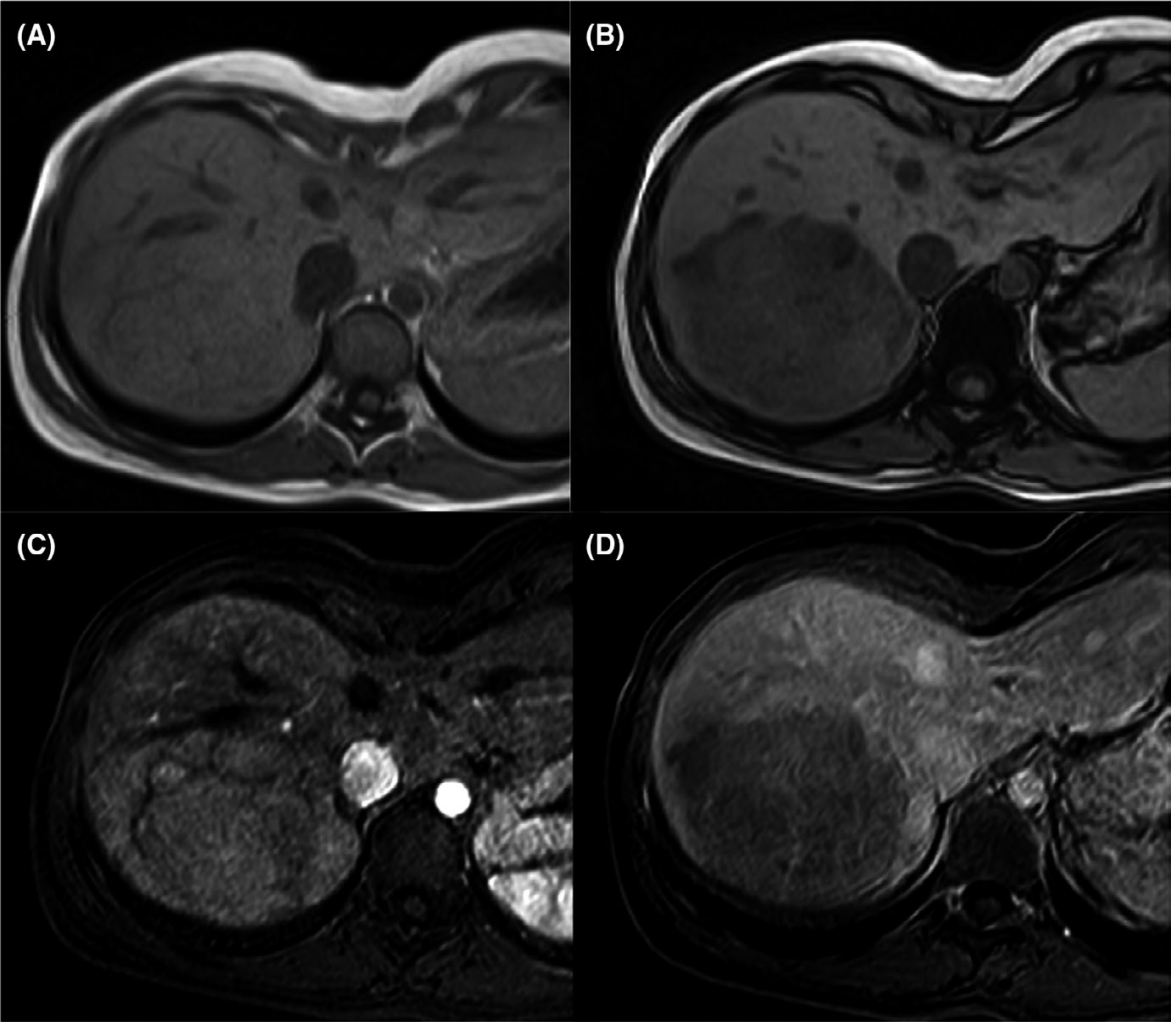
H‐HCA in a 23‐year‐old woman. MR images show a large lesion in the right liver lobe. The MR shows the presence of diffuse fat deposition within the lesion (drop of the signal on opposed‐phase T1‐weighted image B—if compared to in phase image—A). The lesion slightly enhances on hepatic arterial phase (C) and shows washout on portal venous phase (D)

Most of H‐HCA is characterized microscopically by the presence of fat. The resulting diffuse and homogeneous drop of signal on opposed‐phase T1‐weighted MR images (Figure 2) has a very high specificity (89%‐100%) and high sensitivity (87%‐91%) for the diagnosis of H‐HCA.^30^, ^31^ Other imaging features characterizing H‐HCA include mild hyper‐enhancement on hepatic arterial phase, followed by washout on later phases (Figure 2) and hypointensity on the hepatobiliary phase.

### 4.2 Inflammatory HCA

**FIGURE 3:**
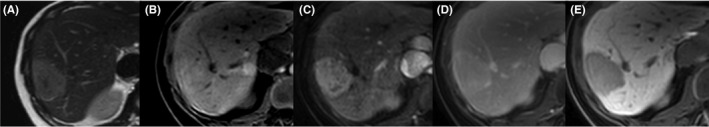
I‐HCA in a 26‐year‐old woman. The lesion is bright on T2‐weighted image (A), is slightly hyperintense on fat‐suppressed T1‐weighted image (B), shows intense and heterogeneous enhancement on arterial phase (C), with persistent enhancement on portal venous phase after gadobenate dimeglumine injection (D). The lesion is hypointense on hepatobiliary phase (E—120 minutes)

I‐HCA is characterized on MRI by strong hyperintensity on T2 images (diffuse or peripheral, “atoll sign”), and persistent enhancement on portal and delayed phases using extracellular MR contrast agents. Combination of these signs has a high specificity (88%‐100%) for classifying inflammatory HCA ^30^, ^31^, ^32^ (Figure 3).

Use of gadoxetic acid as liver‐specific MRI contrast agent may modify the typical persistent enhancement. During transitional phase (3‐5 minutes) vascular structures including dilated sinusoids within the tumour appear hypointense due to the rapid clearance of the gadoxetic acid from the vascular pool.^33^ A study from Ba‐Ssalamah et al showed that while 95% of inflammatory HCA presented with persistent enhancement on the portal phase after gadoxetic acid injection, only 48% showed persistent enhancement during the transitional phase.^34^ Hence persistent enhancement should be only evaluated on portal venous phase when gadoxetic acid is used as contrast agent for MRI, while lesion hypointensity during transitional phase should not exclude the diagnosis of I‐HCA.

In the hepatobiliary phase, most I‐HCA are hypointense (Figure 3). Nevertheless, around one third of them show an iso‐ or hyperintense signal relative to the liver.^34^, ^35^, ^36^ As I‐HCA frequently develop in a steatotic liver and show spontaneous T1 hyperintensity, the reduced intensity of the background parenchyma (due to steatosis) on fat saturated sequences combined with their spontaneous hyperintensity on T1 can explain the lesion iso‐hyperintensity in the hepatobiliary phase.^37^ Therefore, a quantitative approach based on liver‐to‐lesion contrast enhancement ratio (LLCER), helps identify the real contrast uptake in the hepatobiliary phase.^35^ Interestingly, in a recent study, 100% of I‐HCA had a negative (<0%) LLCER whereas 86% of beta‐catenin activated HCA (β‐HCA) had a positive (>0%) LLCER.^37^ The latter may be explained by the conserved expression of OATP (organic‐anion‐transporting polypeptide) in the b‐HCA.

### 4.3 Beta‐catenin activated HCA—Sonic hedgehog activated HCA—Unclassified HCA

**FIGURE 4:**
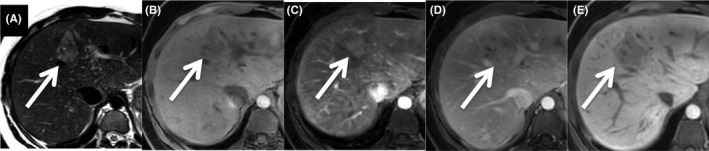
b^ex3^HCA in a 22‐year‐old woman. The lesion of the segment IV of the liver (arrows) is hyperintense with a heterogeneous appearance on T2‐weighted image (A). The lesion is slightly hypointense on fat‐suppressed T1‐weighted image (B), slightly and heterogeneously hyperenhanced on arterial phase (C) and heterogeneously isointense on portal venous phase (D) after gadobenate dimeglumine injection. The lesion is visually hypointense on hepatobiliary phase (E—120 minutes) if compared to the background liver. A quantitative approach using LLCER helps point out the contrast uptake on hepatobiliary phase (LLCER measured at 18.6%). Diagnosis of b^ex3^HCA activated hepatocellular adenoma was confirmed after resection

At present, it is not possible to accurately differentiate b‐HCA, sh‐HCA and U‐HCA subtypes with imaging. Differently from I‐HCA, b‐HCA develop mostly in non steatotic livers.^37^ b‐HCA have initially been reported to be heterogeneous on all MR sequences,^30^ sometimes with necrotic portions but without fat components, typically hypervascular with variable washout appearance.^30^, ^34^ These features are not sufficiently accurate. Practically, the diagnosis of b‐HCA should be considered when a lesion that does not meet imaging criteria of focal nodular hyperplasia shows iso‐ to hyperintensity on the hepatobiliary phase or a positive LLCER between unenhanced and hepatobiliary phase (Figure 4), a heterogeneous appearance on T1‐weighted images and a vague defined scar or heterogeneous appearance on T2‐weighted images.^34^, ^36^, ^37^, ^38^, ^39^ A recent study also suggested the potential added value of low ADC value for the diagnosis of b‐HCA.^40^ These preliminary data need to be validated in larger cohorts. Moreover an evident limitation of these studies is the absence of distinction between exon 3 and exon 7,8 b‐HCA subtypes.

The combined form of I‐HCA and exon 3 or exon 7,8 b‐HCA (b^ex3^IHCA or b^ex7,8^IHCA), are likely to show the same imaging appearance as I‐HCA.^30^, ^41^ Therefore, differentiating between a pure I‐HCA and a b‐IHCA is a new diagnostic challenge in imaging.

Finally, sh‐HCA and U‐HCA do not show specific imaging features and are often not distinguishable from hepatocellular carcinoma. Sh‐HCA could be expected to present with haemorrhagic components at imaging,^21^ but this still needs to be explored further.

## 5 BIOPSY

With the continuing knowledge of HCA subtypes and their varying biological behaviour, subtype determination is increasingly important. At this moment only H‐HCA and I‐HCA can be identified reliably based on contrast enhanced MRI. Given the low risk of malignant transformation and the high sensitivity of MRI in correctly diagnosing H‐HCA, these lesions may not have to be biopsied. However, when there is doubt about the subtype on contrast‐enhanced MRI, a biopsy should be performed in the lesion. Additionally, as I‐HCA cannot be differentiated from b^ex3^IHCA or b^ex7,8^IHCA on imaging yet, biopsy may also be performed in case of an I‐HCA to determine whether the lesion has an additional exon 3 or exon 7,8 mutation. The value of biopsy to identify HCA subtypes at risk for malignancy or haemorrhage is becoming a pivotal step in the management of this rare disease.

## 6 FOLLOW‐UP OF HCA ‐ WHEN TO TREAT?

Treatment of HCA should be reserved for patients with a high risk of haemorrhage and development of HCC. Subgroups at risk are men with HCA, patients with β‐(I)HCA, and patients with HCA showing progressive growth.^13^, ^21^, ^42^ Additionally, the 2016 EASL guideline advised to implement lifestyle changes for all female patients with HCA, irrespective of baseline diameter.^11^ The most important lifestyle change is cessation of oral contraceptives, as it has been shown even in the 1970s that this can lead to regression of HCA.^43^ In the past decade, several studies have also shown that weight loss may lead to regression of HCA in obese patients.^4^, ^44^ The EASL guideline states to perform a surgical resection if HCA exceed 5 centimetres six months after implementation of these lifestyle changes, given the higher risk of complications in HCA > 5cm.^42^, ^45^ Two recent studies however advocate to await the effect of cessation of oral contraceptives for a longer period of time (at least 12 months and longer for larger HCA) as the majority of HCA will regress over time and no complications occurred during follow up in these cohorts.^46^, ^47^

The chance of regression of HCA to <5 cm appears to be lower in H‐HCA as compared to I‐HCA.^47^ Interestingly, a recent study even showed that H‐HCA have a higher rate of progression from <5 cm to >5 cm as compared to I‐HCA, despite a lower and shorter oral contraceptive intake.^48^ A hypothesis may be the varying oestrogen sensitivity of the different HCA subtypes (higher in I‐HCA as compared to H‐HCA), but this has yet to be proven.^21^, ^49^

Haemorrhage is the most frequent complication in HCA, and has been reported in up to 25% of cases.^45^ Not all patients are symptomatic, especially intratumoral haemorrhage may go unnoticed. When lifestyle changes are implemented, treatment is often not required as the hematoma will resorb over time and the tumour will regress. However, in some cases massive bleeding may occur resulting in intraparenchymal haemorrhage, subcapsular hematoma or even hemoperitoneum caused by rupture of the liver capsule. When massive bleeding occurs patients may present with hemodynamically unstable conditions. In the acute phase, conservative management is justified when hemodynamic stabilization can be reached.^50^ In case of persistent hemodynamic instability or active bleeding, transarterial embolization (TAE) is the preferred management.^51^ Liver resection is not advisable in the acute phase as it is associated with increased morbidity and mortality.^50^, ^52^

Pregnancy used to be discouraged in patients with unresected HCA, because of the risk of hormone induced growth and rupture during pregnancy. In 2004, a study was published reporting the mortality risk of ruptured HCA during pregnancy: 44% for the mother and 38% for the foetus.^53^ The majority of cases included in this review dated from the 1970s and 1980s. However, in 2011 it was shown that a large number of patients who were diagnosed with HCA, already had been pregnant and had uneventful pregnancies.^54^ This initiated a study that was recently published, assessing the risk of growth and haemorrhage of HCA <5 cm during pregnancy. In this study, growth occurred in a quarter of cases but no haemorrhage occurred.^55^ No subgroups at risk for growth could be identified in this cohort. Given the fairly high proportion of patients with growing HCA, close monitoring during pregnancy with ultrasound is recommended, enabling an intervention in case of progressive growth.^55^ Future research should focus on trying to identify subgroups at risk of pregnancy‐related complications.

In addition, given the sensitivity of HCA to hormones in fertile women, a study was performed questioning whether surveillance of HCA is still required in post‐menopausal women.^56^ The study showed that HCA become smaller after menopause and that routine follow‐up of small HCA (<5 cm) is not required.^56^

When a patient is diagnosed with HCA, a great number of factors should be taken into account when deciding whether the patient should undergo a resection or whether a wait‐and‐see policy is legitimized. The most important patient related factors to be considered include sex, age and co‐morbidity. For instance, resection is advised in men given the far higher risk of malignant transformation (estimated 50% in men as compared to 5% in females).^23^, ^42^, ^57^ Additionally, although rare, resection may be advised in patients with HCA and hepatitis B or C infection, given the a priori higher risk of HCC. The key tumour factor that should be considered is the HCA subtype: patients with b‐(I)HCA and sh‐HCA are at greater risk of complications and therefore surgical resection is preferred. When malignancy is suspected on imaging a resection should be performed. A wait‐and‐see policy is legitimized in H‐HCA and I‐HCA without ß‐catenin mutation that show regression with lifestyle changes.

As for every type of liver surgery, the anatomical location of the tumour should be taken into account as well as the quality and volume of the future liver remnant. As a large number of patients with HCA are overweight or obese, the presence and degree of steatosis should be taken into account as these factors impact the perioperative complication‐ and mortality rates of liver resections.^58^ A model combining all these factors would be an ideal solution. A decision curve analysis (DCA) is an example of a model requiring a binary decision, in our case this would be surgery versus wait‐and‐see. We would have to decide how many patients we would be willing to operate although they will not have tumour‐related complications, to avoid one patient getting haemorrhage or malignant transformation. To perform such an analysis we would require detailed information on surgical complications in those treated with a resection and the incidence of malignant transformation and haemorrhage in those treated conservatively. A very large population would be needed to estimate the risks and benefits of both treatment strategies. Unfortunately, considering the low incidence of HCA and the rare indications for surgery, it will be hard to realize a prospective study. Furthermore, the surgical expertise will definitely play a major role in the outcome of such a study. Referring these patients to expert liver centres may offer the best‐case scenario for diagnostic and surgical management at present.

## 7 HOW TO TREAT

Elective surgical resection is the gold standard in the treatment of patients with HCA. Resection of HCA can safely be performed with either an open or laparoscopic approach.^59^ Although laparoscopy may require more advanced surgical skills and is dependent on the size and location of the tumour, it also has great benefits as compared to an open approach, including a reduction in blood loss and a shorter duration of hospital stay.^60^, ^61^

Other treatment methods investigated for the treatment of HCA are transarterial embolization (TAE) and tumour ablation (either radiofrequent ablation or microwave ablation). TAE is a well‐established treatment to use for HCA showing acute haemorrhage with hemodynamic instability,^51^ but recent studies have also investigated its safety and efficacy in the elective treatment of non‐haemorrhaging HCA. TAE appears to be a safe and can lead to size reduction of HCA, although its effect is difficult to distinguish from the ongoing effect of cessation of oral contraceptives.^62^, ^63^ Additionally, the effect TAE has on the risk of malignant transformation is still unclear. Tumour ablation might also be used in the treatment of HCA, but often multiple sessions are required and patients might still have residual HCA despite repetitive treatment.^64^ Both TAE and tumour ablation techniques may only be beneficial in patients with small lesions who are poor surgical candidates.^11^

Liver transplantation has been proposed as a treatment for patients with many widespread HCA (>10, liver adenomatosis). To date, this is no longer considered an indication for liver transplantation.^57^ Liver transplantation is a major procedure and given the organ shortage, it should be reserved for those with histological evidence of malignancy that cannot be treated with liver resection.^65^ The only patients with HCA that might be considered for liver transplantation are men with widespread nonresectable HCA and patients with glycogen storage disease and multiple progressing HCA at risk for malignant transformation.

## 8 FINAL REMARKS

HCA may be considered a rare disease. The small number of patients and the limited epidemiological data pose challenges for research and the clinical development. It is preferable to treat patients with suspected HCA in high volume centres with combined expertise of liver surgeons, hepatologists, radiologists and (molecular) pathologists. To optimize the validity and reliability of future research, it is important to collaborate in (inter)national multicentre consortia to optimize our insights in diagnosis and treatment of this rare disease.
